# The effects of androgen deprivation on working memory and quality of life in prostate cancer patients: The roles of hypothalamic connectivity

**DOI:** 10.1002/cam4.4704

**Published:** 2022-03-22

**Authors:** Shefali Chaudhary, Simon Zhornitsky, Alicia Roy, Christine Summers, Tim Ahles, Chiang‐Shan R. Li, Herta H. Chao

**Affiliations:** ^1^ Department of Psychiatry Yale University School of Medicine New Haven Connecticut USA; ^2^ VA Connecticut Healthcare System West Haven Connecticut USA; ^3^ Department of Psychiatry and Behavioral Sciences Memorial Sloan Kettering Cancer Center New York New York USA; ^4^ Departments of Psychiatry and Neuroscience, Interdepartmental Neuroscience Program Yale University School of Medicine, Wu Tsai Institute, Yale University New Haven Connecticut USA; ^5^ Department of Medicine & Yale Comprehensive Cancer Center Yale University School of Medicine New Haven CT USA

**Keywords:** androgen deprivation therapy, cognition, hypothalamus, prostate cancer, quality of life, resting state fMRI

## Abstract

**Background:**

Androgen deprivation therapy (ADT) has been associated with adverse effects on the brain. ADT alters testosterone levels via its action on the hypothalamus‐pituitary‐gonadal axis and may influence hypothalamic functions. Given the wide regional connectivity of the hypothalamus and its role in regulating cognition and behavior, we assessed the effects of ADT on hypothalamic resting state functional connectivity (rsFC) and their cognitive and clinical correlates.

**Methods:**

In a prospective observational study, 22 men with nonmetastatic prostate cancer receiving ADT and 28 patients not receiving ADT (controls), matched in age, years of education, and Montreal Cognitive Assessment score, participated in N‐back task and quality of life (QoL) assessments and brain imaging at baseline and at 6 months. Imaging data were processed with published routines and the results of a group by time flexible factorial analysis were evaluated at a corrected threshold.

**Results:**

ADT and control groups did not differ in N‐back performance or QoL across time points. Relative to controls, patients receiving ADT showed significantly higher hypothalamus‐right mid‐cingulate cortex (MCC) and precentral gyrus (PCG) rsFC during follow‐up versus baseline. Further, the changes in MCC and PCG rsFC were correlated positively with the change in QoL score and 0‐back correct response rate, respectively, in patients with undergoing ADT.

**Conclusion:**

Six‐month ADT affects hypothalamic functional connectivity with brain regions critical to cognitive motor and affective functions. Elevated hypothalamic MCC and PCG connectivity likely serve to functionally compensate for the effects of ADT and sustain attention and overall QoL. The longer‐term effects of ADT remain to be investigated.

## Graphical abstract

Androgen deprivation therapy (ADT) may elicit functional brain changes in men with prostate cancer. Increases in hypothalamus resting‐state functional connectivity as a result of androgen deprivation serve to functionally compensate for the effects of ADT and sustain attention and overall quality of life.

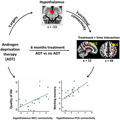

## 1 INTRODUCTION

Prostate cancer is the second most commonly diagnosed and fifth leading cause of death worldwide.^1^ In the United States it stands second among cancer‐related deaths in men. Between 2003 and 2014, 3.1 million new cases of prostate cancer were reported in U.S., with 77% localized, 11% regional, 5% distant, and 7% cases of unknown stage.^2^ Androgen deprivation therapy (ADT) is a widely used treatment of prostate cancer, and nearly 45% of US men diagnosed with prostate cancer receive ADT during the course of their illness.^3^, ^4^ While effective, ADT is associated with cardiovascular and metabolic side effects as well as cognitive impairment, which may impact patients' quality of life.^4^, ^5^, ^6^

The physiological bases of ADT‐associated cognitive deficits have not been fully understood. ADT results in reduction in the level of bioavailable testosterone^7^ through its actions on the hypothalamus‐pituitary‐gonadal (HPG) axis,^8^ which in turn may affect cognition via modulation of hypothalamic neuronal signaling and connectivity.^9^ In particular, with a high density of dopaminergic neurons, the hypothalamus supports motivation, arousal, and affect processing.^10^, ^11^, ^12^, ^13^ It is highly likely that androgen deprivation may elicit hypothalamic dysfunction in patients with prostate cancer.

In animal studies, postnatal castration in rats led to higher dry and cell nuclei weight^14^ and structural changes in the hypothalamus.^15^ Castration relative to sham‐operation attenuated the levels of mRNA precursor of neuromodulin, a membrane‐bound protein involved in regulating neurite growth, neuronal regeneration, and learning and memory,^16^ as well as altered expression of gonadotropic‐releasing hormone in the hypothalamus.^17^, ^18^ Additionally, noradrenaline level of hypothalamic homogenate was elevated post‐gonadectomy in rodents.^19^ Other studies noted reduced γ‐aminobutyric acid turnover^20^ and altered levels of hypothalamic growth hormone post‐castration.^21^ Thus, preclinical evidence supports direct effects of androgen deprivation on hypothalamic structure and function.

In human studies, meta‐analysis associated HPG axis dysregulation, as reflected in flatter diurnal cortisol pattern and blunted awakening cortisol responses, with cognitive impairment in older individuals.^22^ Another meta‐analysis reported that variants of HPG axis‐related genes may differentially affect cognitive performance.^23^ Other studies addressed the influences of sex hormones or hormonal modulators on cognitive performance in patients with mental illnesses.^24^ Additionally, hypothalamic resting state functional connectivity (rsFC) with the subgenual cingulate cortex was reduced in patients with major depression, as compared to healthy subjects.^25^ In healthy individuals, physiological responses to trauma and stress were associated with hypothalamic activity and functional connectivity.^26^ Patients with diffuse axonal injury showed reduction in hypothalamic––basal ganglia and cingulate gyrus rsFC in association with cognitive impairment.^27^ In rodents the formation of object recognition memory was regulated via hypothalamic circuits.^28^ These findings implicate the hypothalamus in learning and memory through mechanisms beyond those involved in energy homeostasis.^29^ A recent work observed overall increases in rsFC in patients receiving ADT as compared to controls, although the study did not specifically examined the hypothalamus or the links to cognitive functions.^30^ A ^18^F‐fluorodeoxyglucose‐positron emission tomography study noted hypothalamic hypometabolism in association with depression, anxiety, and aggression post‐ADT.^31^ No other studies to our knowledge have investigated hypothalamic dysfunction in association with cognitive deficits elicited by ADT.

Brain is known to be organized in functional networks to support cognition and behavior, and many previous studies have shown altered regional rsFC or other network metrics in relation to cognitive or behavioral impairment in cancer patients.^32^, ^33^, ^34^, ^35^, ^36^ In the present study, we evaluated the effects of ADT on hypothalamic rsFC in prostate cancer patients who received ADT and those who did not receive ADT (CON) in a longitudinal setting. Further, we assessed whether or how these changes may relate to cognitive functions and quality of life. We hypothesized that, first, ADT would lead to longitudinal changes in hypothalamus connectivity as compared with CON, as evidenced in a significant treatment (ADT/CON) × time (baseline/follow‐up) interaction effect. Second, ADT would lead to working memory deficits and deterioration in quality of life. Finally, changes (follow‐up vs. baseline) in hypothalamus connectivity would correlate with changes in working memory performance and quality of life in patients receiving ADT.

## 2 MATERIALS AND METHODS

### 2.1 Participants and clinical profiles

Patients were recruited from the Medical Oncology and Urology Clinics at the West Haven VA Connecticut Healthcare System. Patients with biopsy‐proven prostate adenocarcinoma between the age of 55 and 75 years were identified. To limit confounding effects from significant cancer burden, all participants underwent full staging scans and did not have any evidence for distant metastases. The decision on treatment with or without ADT was independent of participation in the study and followed current National Comprehensive Cancer Network and American Urological Society practice guidelines. All men who were prescribed ADT as adjuvant treatment or because of biochemical recurrence were approached for participation. ADT consisted of medical castration with an LH‐RH agonist (Goserelin or Leuprolide) subcutaneously for 6 months, after a lead‐in period with bicalutamide 50 mg daily. Patients with nonmetastatic prostate cancer who had never been treated with ADT participated as controls (CON). For both ADT and CON, exclusion criteria were: Eastern Cooperative Oncology Group Performance Status>1; active second malignancy; significant cardiovascular, liver, renal, or neurological disease; any investigational drugs or contraindications, including claustrophobia, for MRI; current substance (except nicotine) use disorders (use of illicit substances were verified by a urine test); history of Axis I psychiatric illness; history of traumatic brain injury or concussions causing loss of consciousness. All participants underwent a health questionnaire interview to ensure eligibility for fMRI. Participants who had a prostatectomy were at least 3 months from their surgery and had fully recovered from anesthesia before study entry. Participants who were to receive radiation to the prostate underwent baseline assessment and MR scan before starting any treatment and had to be fully recovered from any acute side effects of radiation at the time of their follow‐up assessments. In addition to measuring serum testosterone and prostate‐specific antigen (PSA) as part of their routine bloodwork at every assessment, all participants underwent determination of other hormonal (e.g., cortisol and thyroid hormone) levels that could potentially affect cognitive function.

The study was approved by the Human Investigation Committee at both the West Haven VA and Yale University School of Medicine. All participants offered a written consent prior to the study.

### 2.2 Study procedures and assessment of working memory and quality of life

All participants underwent clinical and cognitive assessment as well as MR scans at baseline and at 6‐month follow‐up.

**FIGURE 1:**
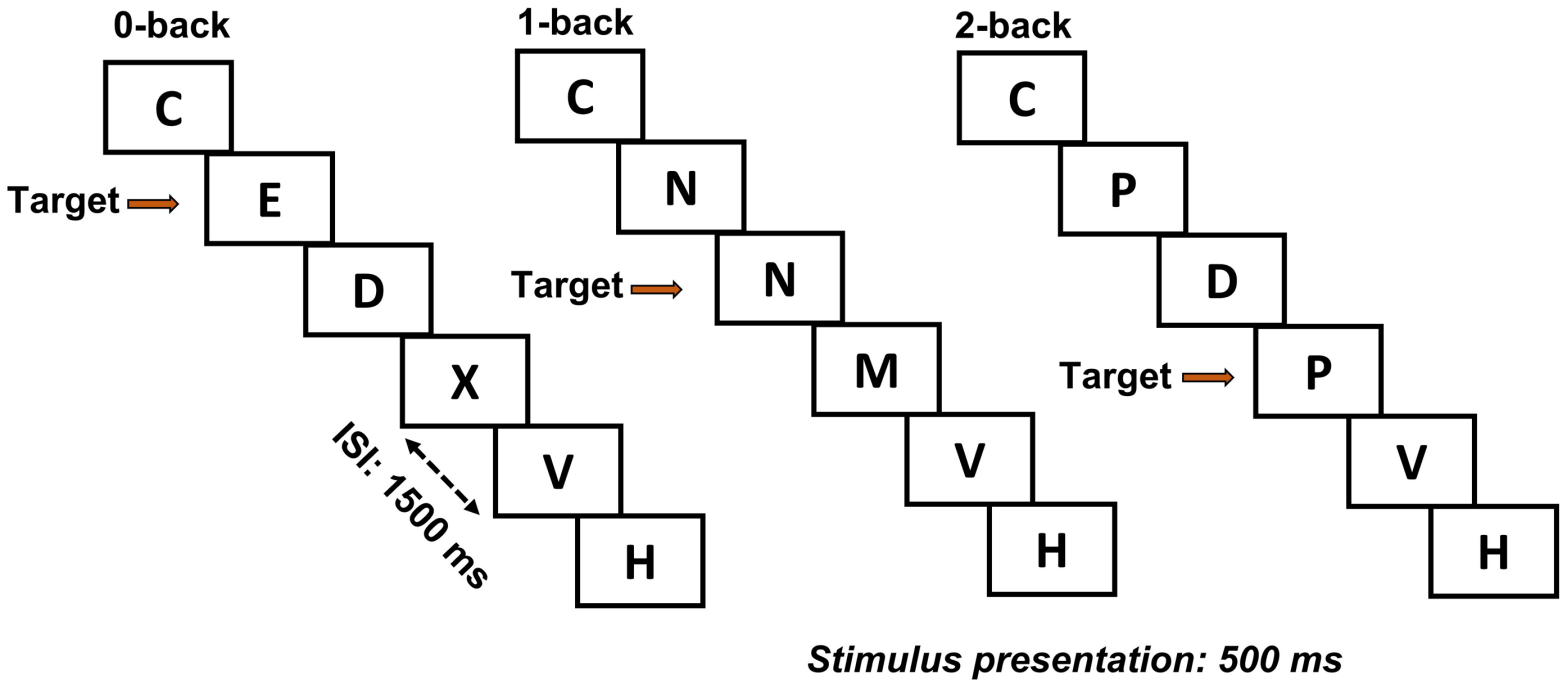
N‐back working memory task. A stream of 15 phonologically distinct letters appears in sequence each for a duration of 500 ms and with an inter‐stimulus‐interval of 1500 ms. There are three different conditions: 0‐, 1‐, and 2‐ back, differing in working memory load. In the 0‐back trials, participants identified a pre‐specified target (e.g., letter “E”); in the 1‐ and 2‐ back trials, there is no fixed target; in contrast, a letter that is the same as the one 1‐ and 2‐time steps back represents the target, respectively. Participants were instructed to response as accurately and as fast as possible. N‐back task was administered at baseline and 6‐month follow‐up outside the scanner. Each subject completed three sessions of the task, with each session containing two each of 0‐, 1‐, and 2‐ back blocks, the order of which was counter‐balanced across sessions. Each block began with an information screen showing the “working memory load” for that block (5 s) and contained 24 trials, with one‐third showing a target. Correct response rate and reaction time, averaged across blocks and sessions, each for 0‐, 1‐, and 2‐back trials, serve as measures of N‐back performance

Working memory is a form of short‐term memory that provides temporary storage and manipulation of information necessary for complex cognitive tasks.^37^ All participants underwent evaluation with the N‐back task, a widely used paradigm to assess working memory (Figure 1).

As a general measure of quality of life (QoL), participants completed the FACT‐P questionnaire at baseline and at 6‐month follow‐up.^38^, ^39^ The cumulative score of FACT‐P subscale scores: physical well‐being (PWB), social well‐being (SWB), emotional well‐being (EWB), functional well‐being (FWB), and prostate cancer subscale (PCS) formed the QoL scores.

### 2.3 Imaging protocol and data analyses

Details of imaging data processing and analyses are described in the Supplement. Briefly, subjects were scanned at baseline and again at 6 months with a 3‐Tesla Siemens Prisma system using a protocol as in our previous studies.^40^ We preprocessed the imaging data and computed whole brain resting state functional connectivity (rsFC) of the hypothalamus for individual participants, following published routines in Statistical Parametric Mapping (SPM12).^13^, ^41^

Nuisance signals unlikely to reflect neural activity were removed using linear regression with the six head motion parameters from realignment, signals from whole brain, ventricular system, white matter, and their first‐order derivatives as covariates.^13^, ^42^ Images were checked for micro‐head motions, followed by “scrubbing” to remove time points affected by head motions^43^ or DVARS(*t*) > 75.^44^ We applied a temporal band‐pass filter (0.009 Hz < f < 0.08 Hz) to the time course to obtain low‐frequency fluctuations and computed the correlation maps to estimate rsFC of the hypothalamus.^45^, ^46^ We employed the hypothalamus mask from the WFU Pick‐Atlas^47^ as the seed, as in an earlier study.^41^ The correlation coefficients between the averaged time course of the hypothalamus seed and time courses of all other brain voxels were computed for each participant. Next, the correlation maps were converted into *z*‐score maps by Fisher's *Z* transform: *z* = 0.5log_e_ [1 + *r*/1 − *r*] (*r* = correlation coefficient) to obtain normally distributed maps.

In view of recent findings of structural brain alterations as a result of cancer‐related treatment,^48^ we also computed the gray matter volumes (GMV) of the hypothalamus. T1‐weighted images were preprocessed and segmented into gray matter, white matter, and cerebrospinal fluid, using standard processing pipeline of voxel‐based morphometry, implemented in Computational Anatomy Toolbox (CAT version 12) (details in Supplementary Methods).^49^ Using the same mask, we extracted hypothalamus GMV for individual participants from the segmented T1‐weighted images.

### 2.4 Statistical analysis

In random‐effects analysis of imaging data, we conducted repeated measures ANOVA with group (ADT/CON) as a between‐subject factor and time point (baseline/6‐month) as a within‐subject factor, using SPM's “flexible factorial analysis” module. The estimated ANOVA model was examined for treatment × time interaction using F‐contrast at *p* < 0.05 FWE‐corrected cluster threshold along with *p* < 0.001 uncorrected voxel threshold, according to current reporting standards.^50^ The rsFC estimates of the clusters identified from the flexible factorial were extracted for further statistical analysis.

In clinical data analyses, we used repeated measures ANOVA to assess differences between follow‐up versus baseline in longitudinal variables across the two groups. Wherever appropriate, between and within group‐changes were assessed using two‐sample and paired *t*‐test, respectively. Correlation between variables was assessed using Pearson's correlation/partial correlation analyses (with covariates). We considered *p* < 0.05 (two‐tailed) as statistically significant.

## 3 RESULTS

### 3.1 Baseline clinical profile of the participants

**FIGURE 2:**
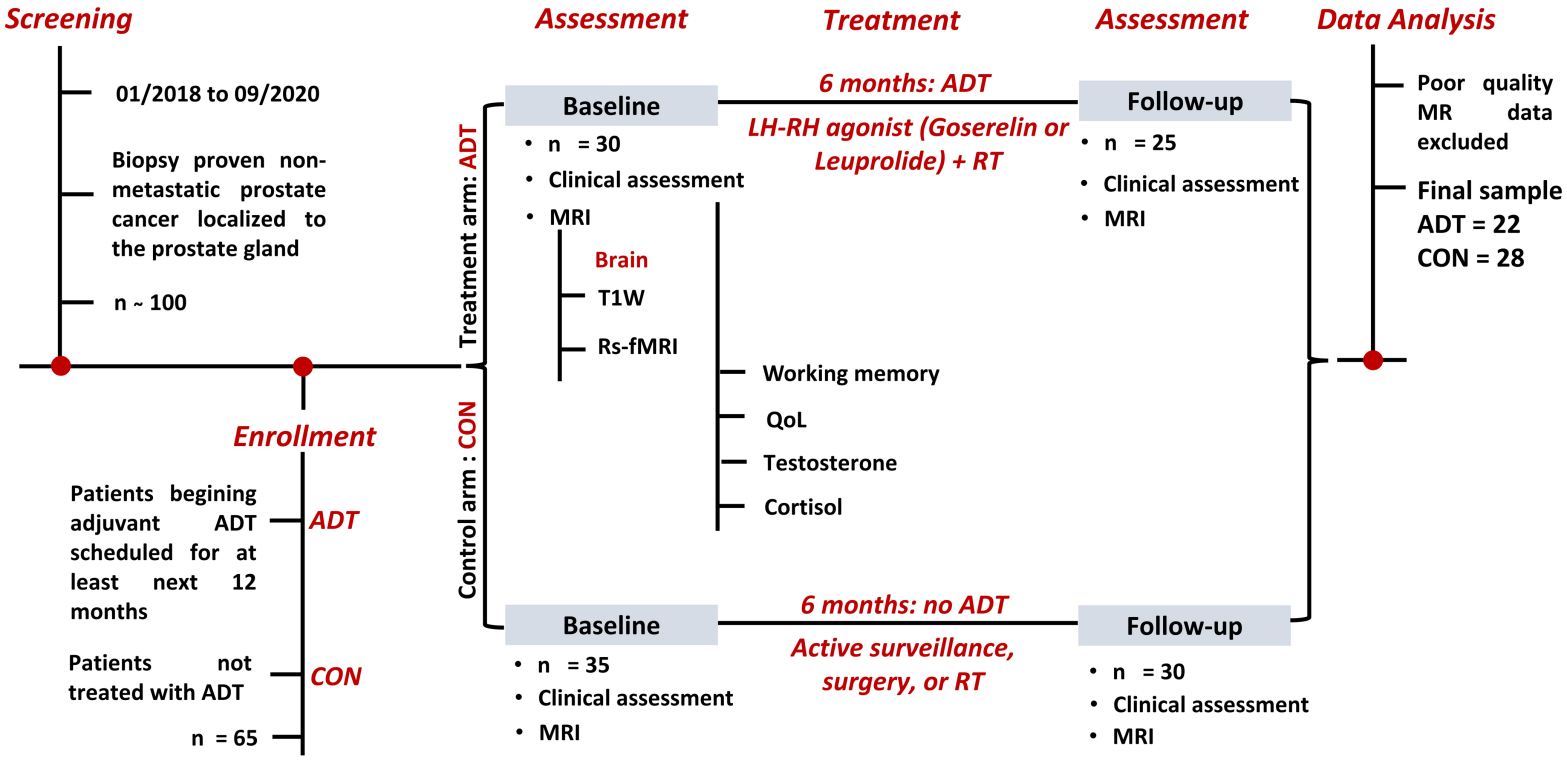
Study timeline. Treatment for the patients followed current guidelines and are independent of the current study. Three patients of the ADT group had previously undergone surgery. ADT, androgen deprivation therapy; CON, control; LH‐RH, luteinizing hormone releasing hormone; MRI, magnetic resonance imaging; QoL, quality of life; rs‐fMRI, resting state functional MRI; RT, radiation therapy; T1W, T1 weighted imaging

Among 100 patients with nonmetastatic prostate cancer, 65 who had never been treated with ADT were enrolled in the study. Thirty patients were scheduled for ADT (ADT group) and 35 patients served as CON. Twenty‐five ADT and 30 CON completed both baseline and follow‐up assessments and MR scans. A total three ADT and two CON were excluded due to excessive head movements during MR scans. Thus, the data from 22 ADT and 28 CON were included in the analyses (Figure 2).

At baseline, the ADT and CON patients did not differ in age, years of education, and MoCA score (Table 1). The testosterone levels were lower in ADT compared to CONs during follow‐up. Thyroid stimulating hormone and T4 thyroid hormone levels were confirmed to be within normal range in all participants (data not shown). The levels of prostate‐specific antigen (PSA, categorized into 6 levels)^51^ were comparable between ADT and CON, both at baseline and at follow‐up (Table 1).

**TABLE 1:** Demographic and clinical characteristics of the patients

	ADT (n = 22)		CON (n = 28)		statistics
Age (years)	66.9 ± 7.2		65.8 ± 5.9		*t*_48_ = 0.6, *p* = 0.547
Edu. (years)	13.3 ± 3.8		14.3 ± 2.4		*t*_48_ = 1.1, *p* = 0.270
MoCA	25.4 ± 2.2		26.6 ± 2.2		*t*_48_ = 1.7, *p* = 0.090^#^
	B	F	B	F	ANOVA*
T level (ng/ml)	3.8 ± 1.7	0.17 ± 0.8	4.29 ± 1.5	3.98 ± 1.5	*F*_1,48_ = 69.4, *p* <0.001
Cortisol (μg/dl)	9.1 ± 4.2	9.1 ± 3.8	8.7 ± 2.4	9.9 ± 2.8	*F*_1,48_ = 1.24, *p* = 0.270
PSA (ng/ml)	<0.01–35.5	<0.01–22	<0.01–13.4	<0.01–15.1	
<2.5	9	21	12	21	0.067^##^
2.6–4.0	1	0	3	0	
4.1–6.0	4	0	4	1	
6.1–10.0	4	0	6	5	
10.1–20.0	1	0	3	1	
>20.0	3	1	0	0	
Quality of life	110 ± 20	113 ± 19	124 ± 17	122 ± 20	*F*_1,48_ = 1.7, *p* = 0.195
PWB	23.5 ± 4.1	22.6 ± 4.4	25.2 ± 2.9	24.6 ± 3.4	*F*_1,48_ = 0.1, *p* = 0.805
SWB	19.6 ± 7.5	21.1 ± 4.4	22.2 ± 4.6	21.8 ± 4.9	*F*_1,48_ = 1.7, *p* = 0.198
EWB	18.8 ± 4.3	20.1 ± 3.7	20.8 ± 2.7	20.9 ± 3.1	*F*_1,48_ = 2.6, *p* = 0.117
FWB	16.1 ± 8.6	18.9 ± 6.1	20.6 ± 6.0	20.1 ± 5.5	*F*_1,48_ = 2.3, *p* = 0.139
PCS	31.8 ± 7.3	30.5 ± 7.3	35.6 ± 6.7	34.8 ± 7.5	*F*_1,48_ = 0.1, *p* = 0.781

*Note*: ^#^covariance analysis with age and years of education accounted for; *treatment × time interaction, ^##^ordered logistic regression, PSA values are represented in range and number of patients in each category; the statistics of the baseline versus follow‐up comparisons are shown in Table S1.

Abbreviations: B, baseline; EWB, emotional well‐being; F, follow‐up; FWB, functional well‐being; MoCA, Montreal Cognitive Assessment; PCS, prostate cancer subscale; PSA, prostate‐specific antigen; PWB, Physical well‐being; SWB, social well‐being; T, testosterone.

### 3.2 N‐back performance

In repeated measures ANOVA, treatment (ADT/CON) × time (baseline/follow‐up) interaction was not significant for any of the N‐back performance metrics (Table 2). Correct response rate (number of correct responses/number of responses) decreased and reaction time of correct trials increased with increasing working memory load for both ADT and CONs. At baseline, N‐back performance was comparable between ADT and CON. During follow‐up, 1‐back correct response rate was higher in CON (91.2 ± 16.4%) as compared to ADT (76.8 ± 24.6%; *t*_48_ = 2.5, *p* = 0.016). The difference remained marginally significant after controlling for baseline age, years of education, and MoCA score (*t*_45_ = 2.0, *p* = 0.052). Other performance metrics were comparable between groups at baseline and follow‐up.

**TABLE 2:** N‐back task performance at baseline and 6‐month follow‐up

	ADT_B	ADT_F	CON_B	CON_F	*p*‐value*
Correct response rate (%)					
0‐back	97.6 ± 5.5	96.8 ± 5.7	99.7 ± 0.7	97.7 ± 5.1	0.445
1‐back	83.5 ± 12.3	76.8 ± 24.6	89.7 ± 14.4	91.2 ± 16.4	0.197
2‐back	62.2 ± 18.2	57.5 ± 18.9	68.2 ± 19.8	66.2 ± 21	0.585
Reaction time of correct trials (ms)					
0‐back	533 ± 105	546 ± 68	512 ± 101	513 ± 81	0.605
1‐back	642 ± 131	670 ± 120	623 ± 168	642 ± 152	0.803
2‐back	775 ± 202	783 ± 209	712 ± 177	733 ± 153	0.815

*Note*: Values are mean ± SD; B, baseline; F, follow‐up; *p‐value, treatment × time interaction of repeated measures ANOVA. The statistics of the baseline versus follow‐up comparisons are shown in Table S1.

### 3.3 Quality of life scores

In repeated measures ANOVA, QoL did not show a significant treatment (ADT/CON) × time (baseline/follow‐up) interaction (*F*_1,48_ = 1.73, *p* = 0.195) (Table 1). At baseline, QoL in ADT was significantly worse compared to CON (two‐sample *t*‐test: *t*_48_ = 2.85, *p* = 0.006). However, no significant difference was observed at follow‐up (*t*_48_ = 1.59, *p* = 0.117). Change in QoL score was not significant in CON (paired *t*‐test: *t*_27_ = 0.86, *p* = 0.397) or ADT (*t*_21_ = 0.95, *p* = 0.350) from baseline to follow‐up. Treatment × time interaction was not significant for QoL total or any of the sub‐scores, either (Table 1). Baseline EWB (two‐sample *t‐*test: *t*_48_ = 2.12, *p* = 0.039) and FWB (*t*_48_ = 2.18, *p* = 0.035) were worse in ADT versus CON. However, none of the group difference in follow‐up scores or changes in scores from baseline to follow‐up were significant in ADT or in CON (Table S1).

### 3.4 Hypothalamus GMV

The treatment × time interaction of repeated measures ANOVA was not significant for hypothalamus GMV (*F*_1,48_ = 0.28, *p* = 0.598). Hypothalamus GMV was comparable between the ADT and CON at baseline (two‐sample *t*‐test: *t*_48_ = 0.11, *p* = 0.913) and follow‐up (*t*_48_ = 0.21, *p* = 0.832). The change at follow‐up from baseline in hypothalamus GMV was not significant in ADT (paired *t*‐test: *t*_21_ = 1.72, *p* = 0.099) or CON (*t*_27_ = 0.73, *p* = 0.469).

### 3.5 Hypothalamic resting state functional connectivity (rsFC)

**FIGURE 3:**
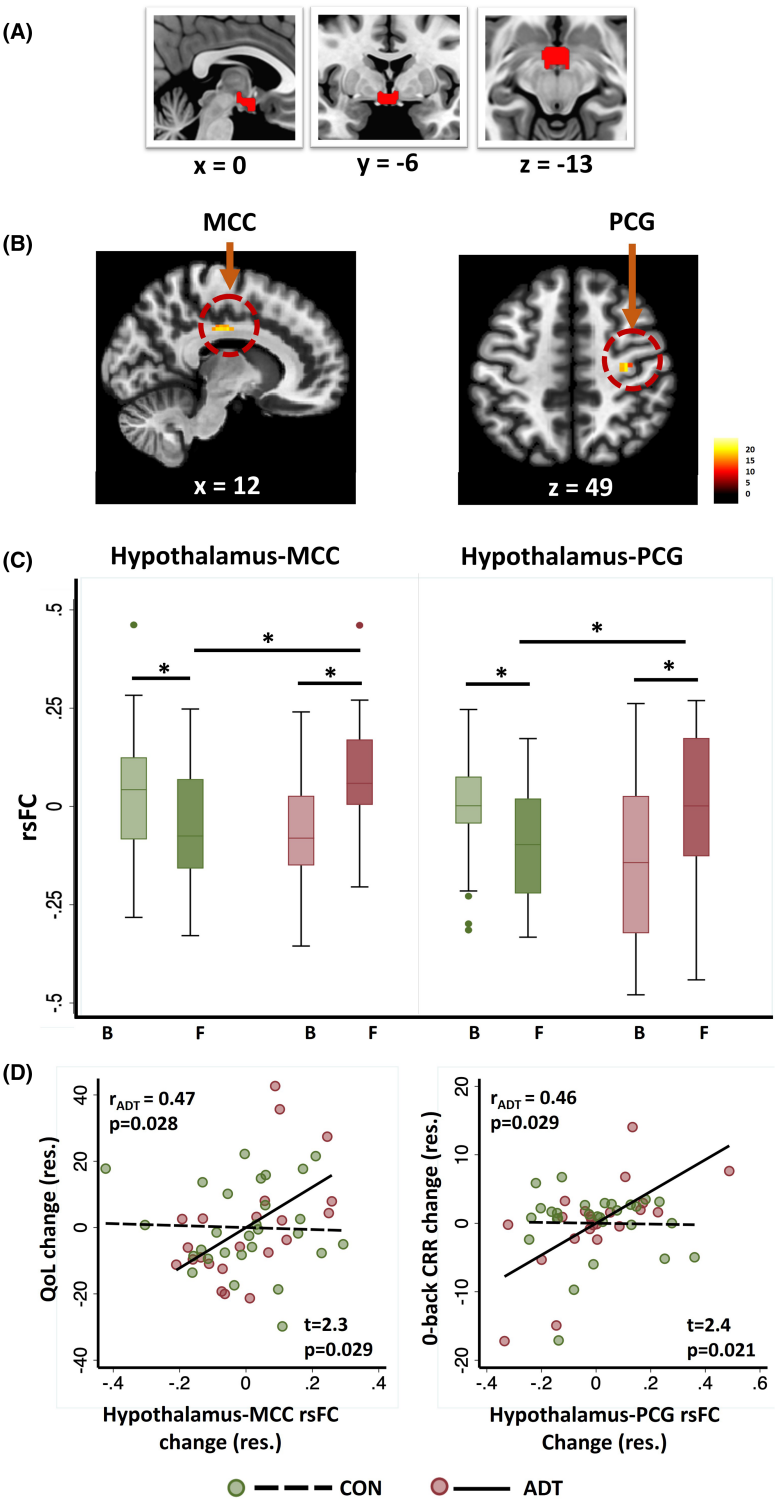
Altered hypothalamic connectivity following ADT. (A) Hypothalamus seed in sagittal, coronal, and axial sections; (B) Clusters showing significant treatment × time interaction in hypothalamus connectivity include the right middle cingulate cortex (MCC) and precentral gyrus (PCG); (C) β estimates of MCC and PCG rsFC in CON and ADT; *p < 0.05, post hoc planned comparisons; (D) Pearson's partial correlation between changes in hypothalamus‐MCC rsFC and in QoL from baseline (B) to follow‐up (F) and between changes in hypothalamus PCG rsFC and 0‐back correct response rate. Each data point represents one subject in the ADT (red) and CON (green) group. The *t* and *p* values are of the slope test between ADT and CON. The Pearson's *r* and corresponding *p*‐value are shown for ADT. The correlations were not significant in CON (Table S1). Note that the residuals are plotted here and the covariates in partial correlation included age, years of education, and MoCA score. ADT, patients undergoing androgen deprivation therapy; B, baseline; CON, control patients; CRR, correct response rate (%); F, follow‐up; QoL, quality of life; rsFC, resting state functional connectivity

The hypothalamus seed is shown in Figure 3A. Flexible factorial analysis of hypothalamus‐whole brain rsFC showed significant treatment (ADT/CON) × time (baseline/follow‐up) interaction in a cluster comprising the right precentral gyrus (PCG) and middle cingulate cortex (MCC) (Table 3; Figure 3B). We extracted the β estimates of rsFC and showed in repeated measures ANOVA that the interaction remained significant after controlling for baseline age for both the hypothalamus‐PCG (*F*_1,48_ = 14.5, *p* <0.001) and hypothalamus‐MCC (*F*_1,48_ = 18.3, *p* <0.001) rsFC. Post hoc tests revealed significant change at follow‐up versus baseline in ADT and CON. Baseline hypothalamus‐PCG rsFC was lower (two‐sample *t*‐test: *t*_48_ = 2.7, *p* = 0.011), but hypothalamus‐MCC rsFC was comparable (*t*_48_ = 1.8, *p* = 0.077) in ADT versus CON. At follow‐up both hypothalamus‐PCG (*t*_48_ = 2.3, *p* = 0.024) and hypothalamus‐MCC (*t*_48_ = 2.1, *p* = 0.038) rsFC were higher in ADT versus CON (Figure 3C).

**TABLE 3:** Brain regions with significant treatment (ADT/CON) × time (baseline/follow‐up) interaction in flexible factorial analysis of whole brain hypothalamus rsFC

*x*	*y*	*z*	Cluster size	Peak Z	Hemisphere	Brain area
25	−28	33	128	4.06	R	PCG
30	−21	38		3.64		
12	18	33		3.67		MCC

Abbreviations: MCC, mid‐cingulate cortex; PCG, precentral gyrus.

We evaluated whether these changes in hypothalamic rsFC were related to changes in QoL and N‐back performance from baseline to follow‐up. The follow‐up versus baseline change in hypothalamus‐MCC rsFC was correlated positively with the change in QoL score in the ADT group (*r* = 0.47, *p* = 0.028). Among the QoL sub‐scores, FWB change was observed in positive correlation (*r* = 0.46, *p* = 0.034) with FC change in ADT. The correlations remained significant after controlling for baseline age, years of education, and MoCA score for both QoL (*r* = 0.53, *p* = 0.021) and FWB (*r* = 0.58, *p* = 0.009) (Figure 3D). The change in hypothalamus‐MCC(R) rsFC was not correlated with the change in N‐back performance metrics in ADT or with N‐back metrics or QoL scores in CON (Table S2).

The change (follow‐up––baseline) in hypothalamus‐PCG rsFC was positively correlated with the corresponding change in 0‐back correct response rate in ADT (Pearson regression: *r* = 0.46, *p* = 0.029). The association remained significant (*r* = 0.65, *p* = 0.003) after controlling for baseline age, years of education, and MoCA score (Figure 3D). The change in hypothalamus‐PCG rsFC was not correlated with the change in QoL scores in ADT or with N‐back metrics or QoL scores in CON (Table S2).

**FIGURE 4:**
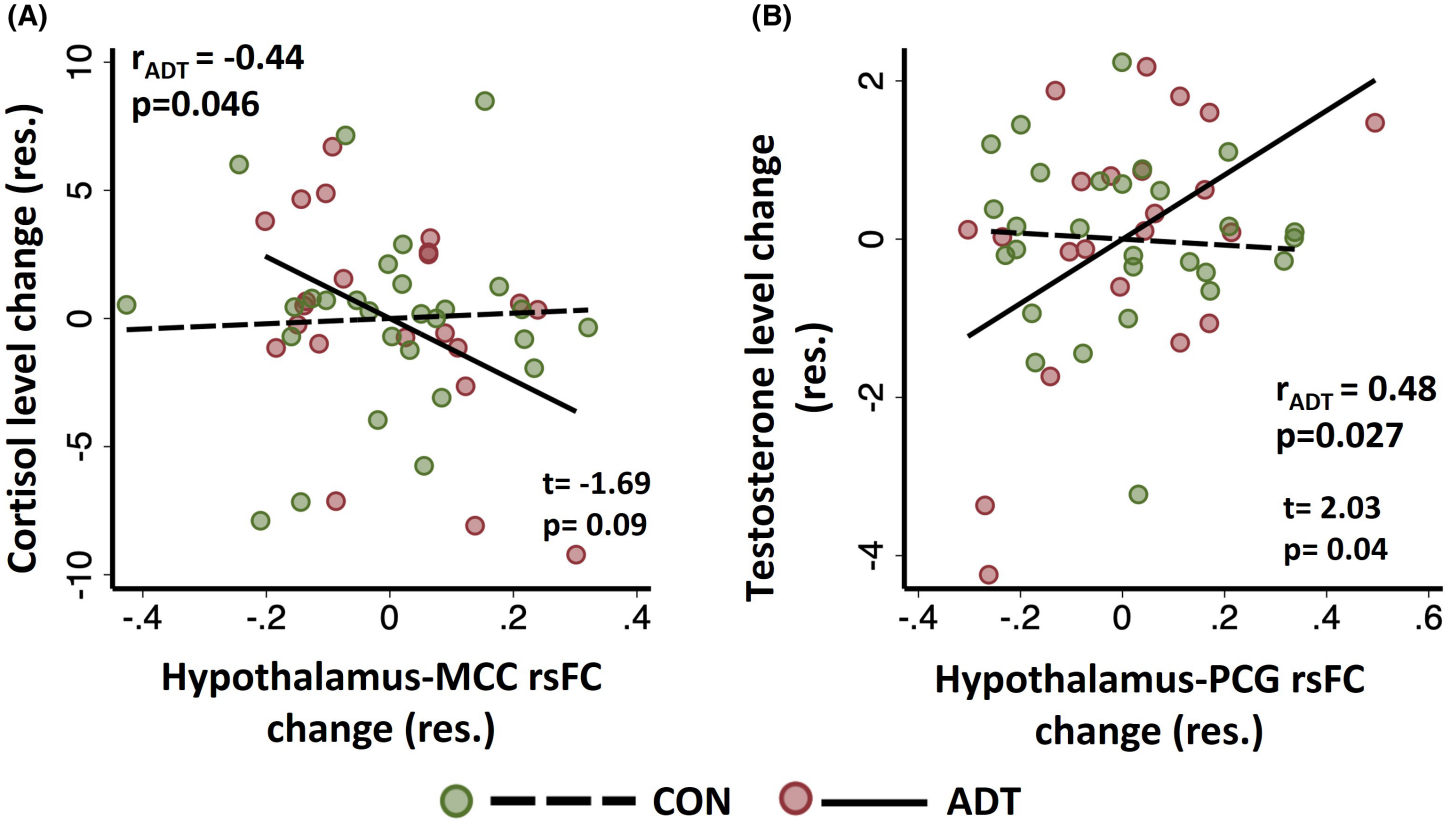
Pearson's partial correlation of (A) the change (follow‐up––baseline) in hypothalamus–– MCC rsFC with the change in cortisol level and (B) the change (follow‐up––baseline) in hypothalamus––PCG rsFC with the change in testosterone level in ADT and CON, with age as covariate. The *t* and *p* values are shown for the slope tests. The Pearson's *r* and corresponding *p*‐value are shown for ADT. The correlations were not significant in CON (Table S2)

We also observed that the changes from baseline to follow‐up in hypothalamus‐MCC rsFC was negatively correlated with the changes in cortisol levels in ADT (Pearson regression: *r* = −0.44, *p* = 0.046) but not in CON (*r* = −0.04, *p* = 0.841). A slope test revealed significant difference in the slopes of the regressions (one‐tailed *t*‐test: *t* = −1.69, *p* = 0.045). Further, the changes from baseline to follow‐up in hypothalamus‐PCG rsFC was positively correlated with the changes in testosterone levels in ADT (*r* = 0.48, *p* = 0.027) but not in CON (*r* = −0.07, *p* = 0.747). A slope test showed significant difference in the slopes of the regressions (*t* = 2.03, *p* = 0.020). The results are shown in Figure 4.

Thus, ADT elicited higher hypothalamic rsFCs at 6 months versus baseline. Although ADT did not alter QoL or N‐back performance on average, individual participants varied in these effects of ADT. ADT patients who showed higher hypothalamic rsFCs also showed better QoL and N‐back performance.

## 4 DISCUSSION

ADT has been associated with physiological, metabolic, and cognitive side effects, which can potentially impact the quality of life (QoL) of prostate cancer patients.^52^ Here, we showed that N‐back working memory and QoL did not appear to be significantly influenced by ADT for a duration of 6 months. With functional brain imaging, we showed elevated hypothalamic resting state functional connectivity (rsFC) with the right‐hemispheric mid‐cingulate cortex (MCC) and precentral gyrus (PCG) in patients undergoing 6 months of ADT, as compared to those who did not receive ADT. Further, the changes in hypothalamic MCC and PCG connectivity were correlated positively with changes in QoL score and 0‐back correct response rate, respectively, in patients on ADT, suggesting a functional compensatory mechanism to maintain well‐being and cognitive function. These findings support the impact of ADT on the brain. It remains to be seen whether ADT for a longer duration may disrupt these adaptive processes and lead to impairment in QoL and cognitive functions.

Hypothalamus regulates the activity of the HPA axis.^53^ Although best known for its role in supporting survival‐related responses, the hypothalamus connects structurally with many brain regions, including the amygdala, cingulate, and other frontal cortical regions,^54^ suggesting that it may partake in cognitive and affective processes beyond immediate physiological need. Here, we observed lower and higher hypothalamus‐MCC/PCG rsFC in CON and ADT, respectively, at follow‐up relative to baseline, suggesting the impact of ADT on hypothalamic rsFCs with cortical structures critical for affective and cognitive motor control.

ADT for 6 months did not appear to alter QoL and N‐back performance metrics, consistent with earlier studies.^4^, ^55^ A possible explanation would be that the changes in hypothalamic rsFCs serve to functionally compensate for the effects of ADT. Indeed, we observed in ADT elevated hypothalamus‐MCC rsFC in positive correlation with QoL, especially with the FWB subscore, suggesting that stronger connectivity maintains better functional well‐being in ADT patients. Note that this correlation was not observed in CON and a direct comparison showed significant differences between ADT and CON in the slope of the regressions. That is, those ADT patients who demonstrated an increase in hypothalamus‐MCC rsFC fared better in QoL. We also observed a negative correlation between the changes in morning cortisol level and hypothalamus‐MCC rsFC in ADT, although the cortisol levels were not significantly altered post‐ADT. Cortisol levels undergo adaptive short‐term surge during stress, and elevate in response to prolonged stress.^56^, ^57^ Thus, the negative correlation of hypothalamus‐MCC rsFC with cortisol levels may reflect a physiological mechanism to regulate stress. In imaging studies of adults with prior psychological trauma, higher cortisol level was associated with smaller right MCC GMV.^58^ During challenges with traumatic imagery, patients with post‐traumatic stress disorder showed higher cingulate activity, as compared to controls.^59^ The observation of altered hypothalamus connectivity with the MCC, a limbic motor region, in negative and positive correlation each with changes in cortisol level and QoL is consistent with functional compensatory hypothalamic response to stress and/or ADT.

Changes in hypothalamus‐right PCG rsFC and 0‐back correct response rate were positively correlated in ADT. Note that right‐ and left‐hemispheric PCG (motor cortex) are connected via the corpus callosum and can be functionally antagonistic.^60^ The right PCG serves to inhibit the left PCG and vice versa during cognitive motor performance. Thus, this finding suggests that less hypothalamic rsFC with the right PCG diminish the inhibition of the left PCG and expedite RT during the 0‐back trials. Speedier RT indicates better attention,^61^ and this finding is consistent with previous reports of the roles of hypothalamus‐frontal cortical connectivity in regulating cognitive processes.^62^ Likewise, it remains to be investigated whether this compensatory process may continue to be adaptive with longer duration of ADT, considering earlier reports of impairment in motor and physical performance in prostate cancer patients undergoing ADT longer than 6 months.^63^, ^64^

### 4.1 Limitations of the study, other considerations, and conclusions

A number of limitations need to be considered. First, the study included a small sample size, and the results would need to be replicated. On the other hand, we wish to emphasize that the imaging results on hypothalamic rsFCs were obtained at a corrected threshold and would likely be robust. Second, working memory represents one aspect of cognitive functions. Studies are needed to employ a more comprehensive battery of neuropsychological tests and other neural metrics to fully investigate potential cognitive dysfunction in prostate cancer patients receiving ADT.^65^ Finally, as patients may undergo ADT for a longer duration, the current findings should be considered as specific to patients with only 6 months of exposure to ADT.

Cancer‐related treatments have been effective in prolonging life but also met with a wide range of side effects in cancer patients.^66^ Understanding these side effects and investigating physiological and life‐style factors to mitigate these side effects should constitute an important focus of cancer survivorship research.^67^, ^68^, ^69^

To conclude, androgen deprivation for 6 months did not lead to significant changes in N‐back working memory or quality of life. This may result from compensatory changes in hypothalamic functional connectivity with cortical regions. These findings support hypothalamus as a critical target to investigate the influences of ADT on cognitive and affective function in prostate cancer patients. In particular, it remains to be seen whether these compensatory processes would continue beyond 6 months of ADT.

## CONFLICT OF INTEREST

The authors have no conflict of interest to disclose.

## AUTHOR CONTRIBUTIONS

Shefali Chaudhary: data curation, data analysis, writing of the original draft, and editing. Simon Zhornitsky: data acquisition, manuscript editing. Alicia Roy: project administration, data acquisition. Christine Summers: project administration, data acquisition. Tim Ahles: conceptualization, methodology, and manuscript editing. Chiang‐Shan R. Li: conceptualization, methodology, data analyses, and manuscript editing. Herta H. Chao: conceptualization, methodology, manuscript editing, and overall supervision of the study. All authors read and approved the final version of the manuscript.

## ETHICS STATEMENT

The study was approved by the Human Investigation Committee at both the West Haven VA and Yale University School of Medicine. All participants offered a written consent prior to the study.

## Supporting information


Appendix S1

## Data Availability

The data would be available from the corresponding author upon reasonable request.
