# Supplementary material for: The effects of androgen deprivation on working memory and quality of life in prostate cancer patients: The roles of hypothalamic connectivity
Source: Cancer Med. 2022 Mar 22;11(18):3425–36. doi: 10.1002/cam4.4704 (PMC9487881; doi:10.1002/cam4.4704)
Supplement: Supplementary file 1 — Appendix S1 [file CAM4-11-3425-s001.docx]

**Supplement**

Chaudhary et al. The Effects of Androgen Deprivation on Working Memory and Quality of Life in Prostate Cancer Patients: The Roles of Hypothalamic Connectivity

**Materials and Methods**

**Imaging Protocol**

Subjects were scanned at baseline and again at 6 months. Brain imaging was performed on a 3-Tesla Siemens Trio TIM system equipped with a 32-channel head coil. Scanning used the conventional T1-weighted spin echo sagittal anatomical images for slice localization. Next, anatomical images of the functional slice localization were acquired using spin echo imaging in the axial plane parallel to the AC–PC line with TR = 1900ms, TE = 2.52ms, bandwidth = 170 Hz/pixel, flip angle = 9°, field of view = 250 × 250 mm, matrix = 256 × 250 mm, 176 slices with slice thickness = 1 mm and no gap. Resting state functional, blood oxygen level-dependent (BOLD) signals with participants in eyes-closed condition, were then acquired with a single-shot gradient echo echoplanar imaging (EPI) sequence in 51 axial slices parallel to the AC–PC line covering the whole brain using TR = 1,000ms, TE = 30ms, bandwidth = 2290 Hz/pixel, flip angle = 62°, field of view = 210 × 210 mm, matrix = 84 × 84, slice thickness = 2.5 mm without gap, and total acquisition time = 10 m.

**Imaging Data Analysis**

**Resting state functional connectivity**

Data were analyzed with Statistical Parametric Mapping (SPM12). Standard preprocessing pipeline was applied on functional images after first discarding the images corresponding to initial five TRs to achieve signal steady state equilibrium. Next, the functional images of each subject were slice time corrected to correct for the temporal offset between slices, and then were motion corrected (realigned). Realignment produced a mean functional image which was co-registered with high resolution structural image and segmented for normalization with affine registration followed by nonlinear transformation. The estimated normalization parameters were subsequently applied to the corresponding functional image volumes for each subject. Next, the functional images were normalized to Montreal Neurological Institute (MNI) space with resampled voxel size of (2.5×2.5×2.5) mm^3^ and then, were smoothed with a Gaussian kernel of 6-mm FWHM.

Nuisance signals unlikely to reflect neural activity, were removed using linear regression by including the six motion parameters from realignment, signal from whole brain, ventricular system, white matter, and their first-order derivatives.^1–5^ Next, functional images were checked for micro-head motion (>0.1 mm) as this may lead to spurious correlations in rsFC analysis, followed by “scrubbing” to remove time points affected by head motions, as successfully applied in previous studies using the thresholds of FD(t)>0.5 mm^1,6^ or DVARS(t) >75^1,7^. Before computing the correlation maps to estimate rsFC, we applied a temporal band-pass filter (0.009 Hz<f<0.08 Hz) to the time course to obtain low-frequency fluctuations.^3–5,8,9^

We employed the hypothalamus mask from the WFU Pick-Atlas^10^ as the seed, according to previous study^11^. The correlation coefficients between the averaged time course of the hypothalamus seed and time courses of all other brain voxels were computed for each participant. Next, the correlation maps were converted into z-score maps by Fisher’s Z transform: z = 0.5log_e_ [1+r/1-r] (r=correlation coefficient) to get normally distributed correlation map. In group, random effects analysis we conducted repeated-measures ANOVA with group (ADT/CON) as a between-subject factor and time point (baseline/6 month) as a within-subject factor, using SPM’s ‘flexible factorial analysis’ module. The estimated ANOVA model was examined for treatment × time interaction using F-contrast at p<0.05 FWE-corrected cluster threshold along with p<0.001 uncorrected voxel threshold.^12^ The rsFC estimates of the clusters identified from the flexible factorial were extracted for further statistical analysis.

**Voxel-based morphometry**

The raw 3D T1 images first underwent a manual quality check and then were reoriented to set the origin close to anterior commissure. Further analysis was done using Computational Anatomy Toolbox (CAT version 12) package in Statistical Parametric Mapping or SPM12.^13^

The reoriented T1-images underwent spatial-adaptive non-local means denoising to remove noise while maintaining the edges followed by resampling to accommodate anisotropic spatial resolution or low-resolution images.^14^ The images were then bias corrected followed by an initial affine registration, initial standard unified segmentation and skull-stripping. The brain was then parcellated into right/ left hemisphere, subcortical areas and cerebellum. Further, local adaptive segmentation transformed the local intensity of all tissue classes to correct local intensity variations (under and over estimations). These intensity corrected images were segmented into gray matter (GM), white matter, and cerebrospinal fluid using an adaptive maximum a-posteriori (AMAP) algorithm, which performs segmentation based on an adaptive estimation that models local variations of the parameters (mean/variance) as slowly varying spatial functions and accounts for local intensity variations and inhomogeneities.^15^ AMAP segmentation was refined by applying a partial volume estimation that effectively estimates fraction of each pure tissue type in each voxel ^16^. The segmented and initially registered tissue class maps were normalized to MNI space using template derived from 555 healthy subjects of the IXI-database using the Geodesic Shooting (Large Deformation Diffeomorphic Metric Mapping) normalization procedure.^17^ Normalized GM maps were modulated to obtain absolute volume of GM, corrected for individual brain size. The modulated GM maps were smoothed using a kernel size of FWHM=8 mm.

**Sample size calculation**

We estimated the sample size required to observe a treatment × time interaction effect of hypothalamus rsFC (primary study outcome) on a sample comprising 5 ADT and 5 CON scanned at baseline and at 6 months follow-up, using GLIMMPSE software.^18,19^ Mean hypothalamus connectivity were: -0.18 (ADT _baseline_), 0.06 (ADT _follow-up_), -0.01 (CON _baseline_), -0.12 (CON _follow-up_), with standard deviation in the range of 0.07 to 0.20, and 0.29 the correlation between time points. To estimate the sample size for repeated-measures analysis of variance (ANOVA) of treatment × time interaction, we chose the upper end of variance (i.e., 0.20) and 0.29 correlation between the time-points. With these parameters and for a Type I error rate = 0.05 as well as assuming a balanced design, a sample size of 16 in each group will have a power of 80% to detect treatment × time interaction. This estimation followed from our earlier study, where we observed significant interaction effect on frontal connectivity with 15 patients in each group.^20^ We decided to recruit more patients in order to account for attrition.

**Results**

**Table S1.** Clinical characteristics, quality of life, and working memory of the patients.

|  | ADT (n=22) | | | CON (n=28) | | |
| --- | --- | --- | --- | --- | --- | --- |
|  | **B** | **F** | **p-value** | **B** | **F** | **p-value** |
| T level (ng/ml) | 3.8 ± 1.7 | 0.17 ± 0.8 | <0.001 | 4.29 ± 1.5 | 3.98 ± 1.5 | 0.167 |
| Cortisol (µg/dl) | 9.1 ± 4.2 | 9.1 ± 3.8 | 0.934 | 8.7 ± 2.4 | 9.9 ± 2.8 | 0.056 |
| PSA (ng/ml) | <0.01-35.5 | <0.01-22 |  | <0.01-13.4 | <0.01-15.1 |  |
| <2.5 | 9 | 21 | 0.007 | 12 | 21 | 0.080 |
| 2.6-4.0 | 1 | 0 |  | 3 | 0 |  |
| 4.1-6.0 | 4 | 0 |  | 4 | 1 |  |
| 6.1-10.0 | 4 | 0 |  | 6 | 5 |  |
| 10.1-20.0 | 1 | 0 |  | 3 | 1 |  |
| >20.0 | 3 | 1 |  | 0 | 0 |  |
| Quality of life | 110 ± 20 | 113 ± 19 | 0.350 | 124 ± 17 | 122 ± 20 | 0.397 |
| PWB | 23.5 ± 4.1 | 22.6 ± 4.4 | 0.372 | 25.2 ± 2.9 | 24.6 ± 3.4 | 0.265 |
| SWB | 19.6 ± 7.5 | 21.1 ± 4.4 | 0.289 | 22.2 ± 4.6 | 21.8 ± 4.9 | 0.565 |
| EWB | 18.8 ± 4.3 | 20.1 ± 3.7 | 0.104 | 20.8 ± 2.7 | 20.9 ± 3.1 | 1.000 |
| FWB | 16.1 ± 8.6 | 18.9 ± 6.1 | 0.135 | 20.6 ± 6.0 | 20.1 ± 5.5 | 0.757 |
| PCS | 31.8 ± 7.3 | 30.5 ± 7.3 | 0.281 | 35.6 ± 6.7 | 34.8 ± 7.5 | 0.466 |
| *correct response rate (%)* | | | | | | |
| 0-back | 97.6 ± 5.5 | 96.8 ± 5.7 | 0.597 | 99.7 ± 0.7 | 97.7 ± 5.1 | 0.050 |
| 1-back | 83.5 ± 12.3 | 76.8 ± 24.6 | 0.286 | 89.7 ± 14.4 | 91.2 ± 16.4 | 0.596 |
| 2-back | 62.2 ± 18.2 | 57.5 ± 18.9 | 0.531 | 68.2 ± 19.8 | 66.2 ± 21 | 0.249 |
| *Reaction time of correct trials (ms)* | | | | | | |
| 0-back | 533 ± 105 | 546 ± 68 | 0.582 | 512 ± 101 | 513 ± 81 | 0.933 |
| 1-back | 642 ± 131 | 670 ± 120 | 0.407 | 623 ± 168 | 642 ± 152 | 0.348 |
| 2-back | 775 ± 202 | 783 ± 209 | 0.532 | 712 ± 177 | 733 ± 153 | 0.772 |

Note: values are in mean ±SD, PSA values are represented in range and number of patients in each category; p-values were estimated using paired t-test except for PSA in-which we used χ^2^ test

**Table S2.** Pearson’s correlation between hypothalamus rsFC change (F-B) and corresponding change in QoL and N-back scores

|  | ADT | | CON | |
| --- | --- | --- | --- | --- |
|  | Pearson’s r | p-value | Pearson’s r | p-value |
| Hypothalamus-MCC rsFC (F-B) change | | | | |
| QoL | 0.46 | 0.028 | -0.08 | 0.695 |
| PWB | 0.19 | 0.379 | -0.17 | 0.376 |
| SWB | 0.26 | 0.238 | 0.23 | 0.232 |
| FWB | 0.46 | 0.034 | 0.03 | 0.875 |
| EWB | 0.17 | 0.457 | 0.03 | 0.861 |
| 0-back correct response rate | 0.15 | 0.512 | -0.02 | 0.912 |
| 0-back correct reaction time | -0.15 | 0.495 | -0.17 | 0.380 |
| 1-back correct response rate | 0.10 | 0.645 | -0.11 | 0.585 |
| 1-back correct reaction time | 0.06 | 0.805 | -0.86 | 0.663 |
| 2-back correct response rate | -0.27 | 0.230 | 0.22 | 0.261 |
| 2-back correct reaction time | 0.35 | 0.106 | -0.27 | 0.159 |
| Hypothalamus-PCG rsFC (F-B) change | | | | |
| QoL | -0.11 | 0.638 | -0.18 | 0.368 |
| PWB | 0.19 | 0.385 | -0.10 | 0.601 |
| SWB | -0.28 | 0.199 | 0.05 | 0.786 |
| FWB | -0.07 | 0.770 | -0.18 | 0.353 |
| EWB | 0.23 | 0.302 | 0.02 | 0.934 |
| 0-back correct response rate | 0.46 | 0.029 | -0.09 | 0.657 |
| 0-back correct reaction time | 0.005 | 0.983 | -0.10 | 0.599 |
| 1-back correct response rate | -0.18 | 0.433 | 0.04 | 0.849 |
| 1-back correct reaction time | -0.08 | 0.710 | 0.08 | 0.667 |
| 2-back correct response rate | 0.004 | 0.986 | -0.15 | 0.441 |
| 2-back correct reaction time | 0.008 | 0.971 | 0.221 | 0.258 |

**Supplementary References**

1. Zhang S, Wang W, Zhornitsky S, Li C-SR. Resting State Functional Connectivity of the Lateral and Medial Hypothalamus in Cocaine Dependence: An Exploratory Study. *Front psychiatry*. 2018;9:344. doi:10.3389/fpsyt.2018.00344

2. Rombouts SARB, Stam CJ, Kuijer JPA, Scheltens P, Barkhof F. Identifying confounds to increase specificity during a “no task condition”. Evidence for hippocampal connectivity using fMRI. *Neuroimage*. 2003;20(2):1236-1245. doi:10.1016/S1053-8119(03)00386-0

3. Fox MD, Snyder AZ, Vincent JL, Corbetta M, Van Essen DC, Raichle ME. The human brain is intrinsically organized into dynamic, anticorrelated functional networks. *Proc Natl Acad Sci U S A*. 2005;102(27):9673-9678. doi:10.1073/pnas.0504136102

4. Fair DA, Schlaggar BL, Cohen AL, et al. A method for using blocked and event-related fMRI data to study “resting state” functional connectivity. *Neuroimage*. 2007;35(1):396-405. doi:10.1016/j.neuroimage.2006.11.051

5. Fox MD, Raichle ME. Spontaneous fluctuations in brain activity observed with functional magnetic resonance imaging. *Nat Rev Neurosci*. 2007;8(9):700-711. doi:10.1038/nrn2201

6. Power JD, Barnes KA, Snyder AZ, Schlaggar BL, Petersen SE. Spurious but systematic correlations in functional connectivity MRI networks arise from subject motion. *Neuroimage*. 2012;59(3):2142-2154. doi:https://doi.org/10.1016/j.neuroimage.2011.10.018

7. Li J, Kong R, Liégeois R, et al. Global signal regression strengthens association between resting-state functional connectivity and behavior. *Neuroimage*. 2019;196:126-141. doi:https://doi.org/10.1016/j.neuroimage.2019.04.016

8. Cordes D, Haughton VM, Arfanakis K, et al. Frequencies contributing to functional connectivity in the cerebral cortex in “resting-state” data. *AJNR Am J Neuroradiol*. 2001;22(7):1326-1333.

9. Lowe MJ, Mock BJ, Sorenson JA. Functional connectivity in single and multislice echoplanar imaging using resting-state fluctuations. *Neuroimage*. 1998;7(2):119-132. doi:10.1006/nimg.1997.0315

10. Maldjian JA, Laurienti PJ, Kraft RA, Burdette JH. An automated method for neuroanatomic and cytoarchitectonic atlas-based interrogation of fMRI data sets. *Neuroimage*. 2003;19(3):1233-1239. doi:10.1016/s1053-8119(03)00169-1

11. Le TM, Liao D-L, Ide J, et al. The interrelationship of body mass index with gray matter volume and resting-state functional connectivity of the hypothalamus. *Int J Obes*. 2020;44(5):1097-1107. doi:10.1038/s41366-019-0496-8

12. Kong J, Huang Y, Liu J, et al. Altered functional connectivity between hypothalamus and limbic system in fibromyalgia. *Mol Brain*. 2021;14(1):17. doi:10.1186/s13041-020-00705-2

13. Farokhian F, Beheshti I, Sone D, Matsuda H. Comparing CAT12 and VBM8 for Detecting Brain Morphological Abnormalities in Temporal Lobe Epilepsy. *Front Neurol*. 2017;8(AUG):428. doi:10.3389/fneur.2017.00428

14. Ashburner J, Friston KJ. Unified segmentation. *Neuroimage*. 2005;26(3):839-851. doi:10.1016/j.neuroimage.2005.02.018

15. Rajapakse JC, Giedd JN, Rapoport JL. Statistical approach to segmentation of single-channel cerebral MR images. *IEEE Trans Med Imaging*. 1997;16(2):176-186. doi:10.1109/42.563663

16. Tohka J, Zijdenbos A, Evans A. Fast and robust parameter estimation for statistical partial volume models in brain MRI. *Neuroimage*. 2004;23(1):84-97. doi:10.1016/j.neuroimage.2004.05.007

17. Ashburner J, Friston KJ. Diffeomorphic registration using geodesic shooting and Gauss–Newton optimisation. *Neuroimage*. 2011;55(3):954-967. doi:https://doi.org/10.1016/j.neuroimage.2010.12.049

18. Kreidler SM, Muller KE, Grunwald GK, et al. GLIMMPSE: Online Power Computation for Linear Models with and without a Baseline Covariate. *J Stat Softw*. 2013;54(10):i10. doi:10.18637/jss.v054.i10

19. Guo Y, Logan HL, Glueck DH, Muller KE. Selecting a sample size for studies with repeated measures. *BMC Med Res Methodol*. 2013;13:100. doi:10.1186/1471-2288-13-100

20. Chao HH, Uchio E, Zhang S, et al. Effects of androgen deprivation on brain function in prostate cancer patients - a prospective observational cohort analysis. *BMC Cancer*. 2012;12(1):371. doi:10.1186/1471-2407-12-371
